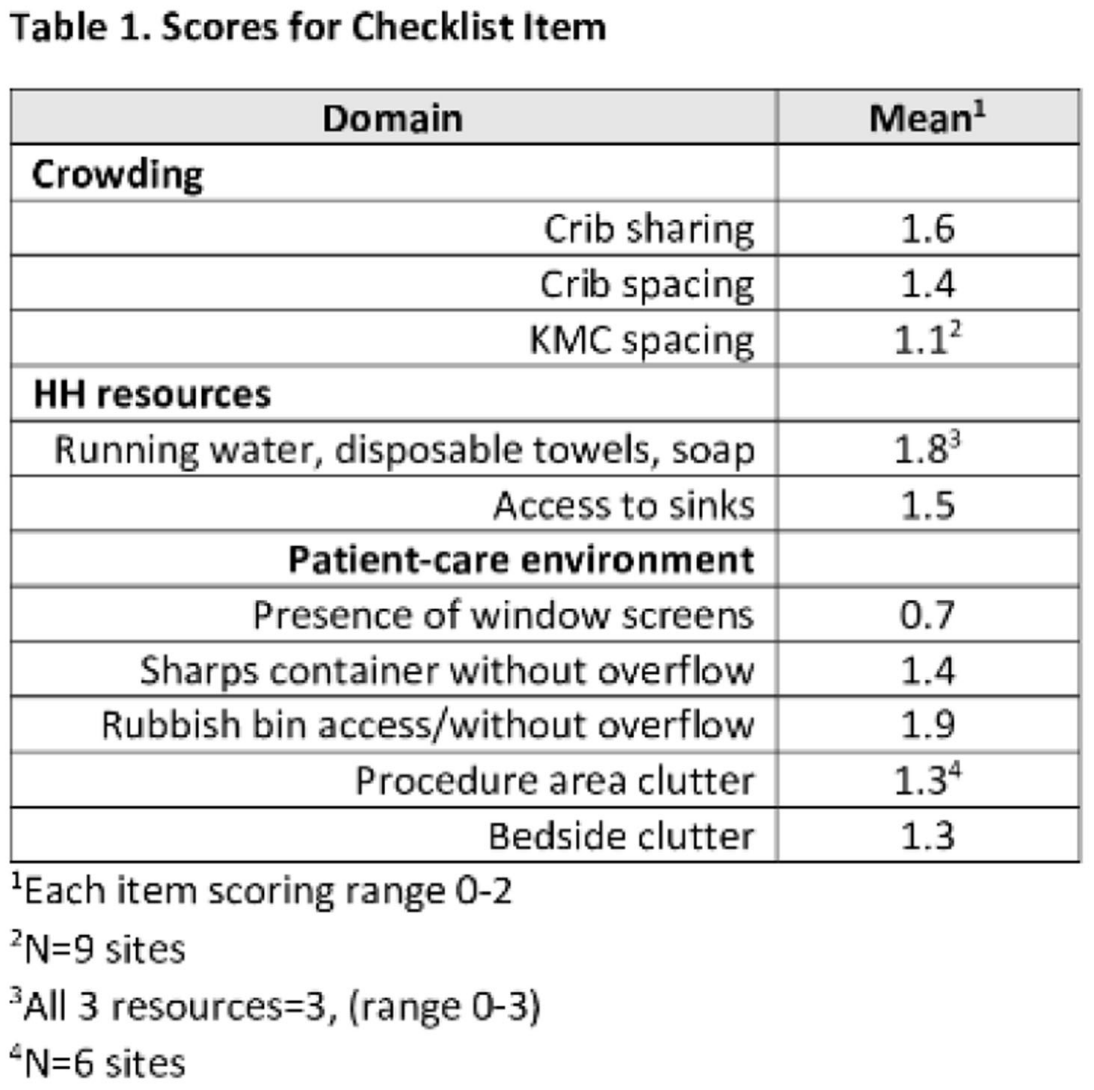# Virtual assessments of infection prevention and control practices in African neonatal facilities: A pilot study

**DOI:** 10.1017/ash.2022.150

**Published:** 2022-05-16

**Authors:** Irene Frantzis, Jack Huebner, Stephanie Levasseur, Aboubacar Sidiki Nabé, Maitry Mahida, Philip Larussa, Wilmot G. James, Lawrence Stanberry, Lisa Saiman

## Abstract

**Background:** Evidence-based infection prevention and control (IPC) practices to reduce healthcare-associated infections in low- and middle-income countries may be difficult to implement due to lack of resources. We pilot-tested the feasibility of virtual assessments of IPC practices in African facilities caring for small and/or sick neonates for opportunities to improve IPC. **Methods:** We created a checklist (in English and French) to assess IPC practices in African facilities caring for small and/or sick neonates **Results:** In total, 10 sites participated in this pilot study. Among them, 3 sites had unreliable Internet connections, and all checklist items could be observed and scored in these videos and photos. The lowest scores occurred for kangaroo mother care (KMC) spacing and presence of screens (Table 1). **Conclusions:** This pilot study demonstrated the feasibility of using virtual assessments of IPC practices. We identified several potentially low-cost opportunities to improve IPC. We are recruiting additional sites to confirm the findings of this pilot study.

**Funding:** Bill and Melinda Gates Foundation

**Disclosures:** None

**Table:**